# EXAMINING THE IMPACT OF FAR-INFRARED TECHNOLOGY ON QUALITY OF LIFE IN OLDER ADULTS

**DOI:** 10.1093/geroni/igac059.2875

**Published:** 2022-12-20

**Authors:** Arianna Balingit, Joshua Guggenheimer

**Affiliations:** St. Catherine University, St.Paul, Minnesota, United States; St. Catherine University, St. Paul, Minnesota, United States

## Abstract

The purpose of this study was to examine the effects of far-infrared heat (FIR) on pain management and quality of life (QOL) in older adults (OA). FIR utilizes a long-wave light that simulates dry sauna-like conditions. Examining the relationship between FIR and pain is important due to the increased prevalence of chronic pain associated with aging and the impact it has on QOL and physical performance. 9 OA completed the study, 8 of whom were women. Our intervention consisted of assigning participants to either a convective (CON) or a convective and far-infrared heat (FIR) group, with convective heat set to 60oC. Participants received 6, 30-minute heat sessions over 3 weeks. Pre- and post-test assessments included physical measures such as range of motion, gait speed, timed up-and-go, and hand grip strength. Additionally, we conducted standardized questionnaires to determine pain severity and interference on daily life, and the impact such has on overall QOL. T-tests were used to compare the groups’ pre- and post-assessment responses. Results indicated that pain severity was significantly reduced (from 3.31 to 2.5, P < 0.05) in the FIR group from pre-to-post, and that pain inference was significantly reduced (from 1.26 to 0.43, P < 0.05) in the CON group from pre-to-post. There were no significant differences from pre-to-post testing for any other measures. These findings suggest that heat therapy was successful in reducing pain over time for OA, but that FIR heat specifically, was not superior to that of convective heat alone.

